# A Nationwide Natural Experiment of e-Health Implementation during the COVID-19 Pandemic in Poland: User Satisfaction and the Ease-of-Use of Remote Physician’s Visits

**DOI:** 10.3390/ijerph19148338

**Published:** 2022-07-08

**Authors:** Mariusz Duplaga

**Affiliations:** Department of Health Promotion and e-Health, Institute of Public Health, Faculty of Health Sciences, Jagiellonian University Medical College, 31-066 Kraków, Poland; mariusz.duplaga@uj.edu.pl

**Keywords:** e-health, telemedicine, remote visit, user satisfaction, ease-of-use, health literacy, e-health literacy, conspiracy beliefs, intent to vaccinate, COVID-19 pandemic

## Abstract

The COVID-19 pandemic resulted in a considerable increase in the use of e-health applications. Shortly after confirmation of the first case of COVID-19 in Poland, the Ministry of Health allowed for the general use of remote physician’s visits (RPVs) as a substitute for traditional visits to the physician’s office. It was estimated that during the first year of the pandemic, as many as 80% of primary care visits were provided remotely, mainly by phone. This study’s main aim was to assess the use of e-health services in the initial phase of the COVID-19 pandemic. Furthermore, the factors related to user satisfaction and positive assessment of the ease-of-use of RPVs were analyzed. The analysis was based on data obtained from a computer-assisted web-based interviewing (CAWI) survey among 2410 adult Internet users in Poland. The questionnaire consisted of 55 items, including a 16-item European Health Literacy Questionnaire, an 8-item e-Health Literacy scale, a set of questions about the use of and experience with e-health services during the pandemic, and items exploring the sociodemographic characteristics of the respondents. Univariate logistic regression models were developed for variables reflecting user satisfaction and the assessment of the ease-of-use of RPVs. The use of RPVs increased during the pandemic by about 200%. Higher health literacy and e-health literacy, older age, higher income, a greater number of e-health services used before the pandemic, and telephone-based remote visits were significantly associated with higher user satisfaction and ease-of-use of RPVs. Respondents using RPVs for renewal of prescriptions were more favorable in assessing satisfaction and ease-of-use. A less positive assessment of satisfaction and ease-of-use was provided by students and vocationally passive persons in comparison to the employed. Finally, the perception of the threat of COVID-19 was associated with higher satisfaction and better assessment of ease-of-use. Persons declaring the intention to be vaccinated against COVID-19 were more likely to be satisfied with remote visits. User satisfaction and the feeling of ease-of-use in the case of remote advice provided by a physician depend on many factors. Significant predictors include selected sociodemographic and economic variables, determinants associated with the perception of the threat of COVID-19, the aims and tools used for the RPVs, and earlier experience with e-health services.

## 1. Introduction

The COVID-19 pandemic led to an abrupt increase in the use of e-health services in many countries. Even in countries with well-developed e-health infrastructures before the pandemic, this growth was significant [1]. Some authors have even used the phrase ‘the dawn of e-health’ [2] to describe the widespread use of e-health services in the first phase of the pandemic. It seems that e-health may have been an important asset in mitigating the epidemic [3,4,5]. As a result, the ability to use e-health solutions was recognized as a social determinant of health [6].

The availability of e-health and telemedicine services in the pre-pandemic period in Poland was relatively limited. Although in 2016, the acts regulating medical professions were updated with statements allowing for the provision of the health services through ‘teleinformatic and communication systems’ [7,8], the actual number of remote interactions between health professionals and patients was rather low. Remote physician’s visits were only reimbursed by the payer in the health care system, the National Health Fund (NHF), in exceptional cases. E-health services could decidedly not be perceived as an alternative to traditional care. However, there was an ongoing effort on the part of the governmental agencies to increase access to such e-health services as Internet Patient Portal, e-prescription, e-confirmation of sick leave, or e-referral for diagnostic procedures or specialized care. Earlier, online booking for the visits to a physician’s office had been provided by many health care institutions. 

The announcement of the state of the epidemic in March 2020 was quickly followed by recommendations from the Ministry of Health (MoH) and the NHF about the possibility of substituting traditional visits to a physician’s office with remote visits carried out by phone or videoteleconferencing (VTC) system. These recommendations, first applied to primary care, were soon extended to specialty ambulatory care [9]. According to the report published in July 2020, under the auspices of the MoH and the NHF, more than 80% of all visits to primary care physicians from the beginning of the COVID-19 pandemic were carried out remotely, mainly by telephone, with a very small percentage based on VTC systems [10]. The level of satisfaction with such remote visits was very high among patients. Later, the enthusiasm for televisits decreased, and the MoH issued additional regulations excluding some situations from remote care. 

Utilizing health services and interacting with health care providers is an element of the capacities covered by health (HL) and e-health literacy (eHL). HL is defined as a set of skills, competencies, and attitudes related to accessing, understanding, appraising, and applying health information for maintaining and promoting own health and that of the closest persons [11]. eHL addresses similar skills and competencies but is focused on health-related information available from electronic sources [12]. HL and eHL have been indicated by many authors as protective factors against the infodemic accompanying the COVID-19 pandemic [13,14,15]. Developing appropriate levels of HL and eHL was also perceived as a way of preparing societies to adopt preventive behaviors to lower the transmission of the new coronavirus [15,16,17]. eHL is usually treated as an indicator of the acceptance and readiness-to-use of e-health applications or at least use of online health information. The interest in the influence of HL on the use of e-health and telemedicine increased substantially during the pandemic [18,19,20,21]. 

The sudden extension of access to e-health services, especially remote physician’s visits, was dictated by the necessity of making health services available to patients during the pandemic threat [3,5]. Unfortunately, this transition was not preceded by the appropriate preparation and training of potential users, either patients or health professionals. Some e-health functionalities, e.g., e-sick leave confirmation, had already been in use before the pandemic. Obligatory e-prescriptions had been introduced in the beginning of 2020, but remote physician’s visits were available before 2020 only to a limited number of citizens, mainly those with access to special health insurance packages offered to corporate clients. Clear guidelines on how to conduct televisits were only issued four months after the announcement of the epidemic status by the Supreme Medical Council [22]. A standard for delivery of remote primary care visits was published by the MoH even later [23]. 

Since the beginning of the COVID-19 pandemic, many authors have addressed the challenges related to the rapid expansion of e-health and telemedicine services. Many reports have analyzed the importance of sociodemographic and economic factors [24,25,26,27,28] or potential disparities in their usage [29,30]. Some authors have also addressed other determinants of e-health usage. For example, Maietti et al. reported the importance of perceived support from the service and an increase in disease self-management among patients with diabetes [31]. In turn, Kong et al. reported that patient portal use among the chronically ill depended on the level of control, the scale of depression, and the status of life satisfaction [32].

The rapid transition from rare to widespread use of e-health and telemedicine services may be perceived as a natural experiment. This term usually defines a situation when the circumstances associated with implementing a specific intervention are not controlled by researchers [33]. Taking advantage of this unique opportunity was the main motivation for the analysis of the reaction of society to the broad introduction of access to e-health services. This study’s main aim was to assess the changes in the use of e-health services in the first year of the COVID-19 pandemic, compared to the pre-pandemic period. Furthermore, the determinants of user satisfaction and ease-of-use of remote physician visits were analyzed. Apart from sociodemographic variables, the roles of HL and eHL, pre-COVID-19 era experience with e-health use, the circumstances of the remote visits, and the perceived health threat of COVID-19, as well as the intention to vaccinate against COVID-19, were assessed.

## 2. Materials and Methods

### 2.1. Survey

The analysis presented in this paper is based on a computer-assisted web-interviewing (CAWI) survey carried out in October 2020 in Poland among a sample of 2410 adult Internet users. Assuming a target population of 28,600,000, a fraction of 0.5, and a confidence level of 0.95, the anticipated sampling error was 2.0%. 

The questionnaire utilized in the survey consisted of 55 items. It included a 16-item European Health Literacy Questionnaire (HLS-EU-Q16) developed by the team of the European Health Literacy Survey Project, an 8-item Polish version of the e-Health Literacy Scale (Pl-eHEALS), a set of questions asking about the use of and experience with e-health services before and during the pandemic, and a set of items exploring the sociodemographic and economic characteristics of the respondents. 

The study was conducted by a poll opinion company, maintaining its own Internet panel of respondents, after receiving the agreement from the Bioethical Committee of the Jagiellonian University (Decision No 1072.6120.99.2020 from 23 April 2020, with amendments). The participants were informed about the study objectives and had to confirm their consent before joining the survey.

### 2.2. Variables

The dependent variables used in the logistic regression models were established based on the responses to items asking about user satisfaction and the ease-of-use of physician’s televisits during the first six months of the COVID-19 pandemic in Poland. The responses were provided on 7-point Likert scales showing the range of opinions from decidedly unfavorable (decidedly unsatisfied or difficult) to decidedly positive (decidedly satisfied or easy to use). The responses were then transformed into a numerical scale from 1 to 7. The variables were dichotomized; responses from 5 to 7 were assigned value ‘1’ and the remaining responses, corresponding with undecided or negative opinions, were assigned value “0”. 

Independent variables used in the regression models included

- Sociodemographic variables: age, gender, place of residence, level of education, marital status, vocational status, and the level of income;
- HL and eHL;
- Variables reflecting the tools used for televisits: telephone, VTC, e-mail;
- Variables reflecting the aims of televisits: symptoms suggesting COVID-19, other acute symptoms, follow-up or exacerbation of the chronic disease, ill children or other family members, renewal of prescriptions;
- The number of different types of e-health services utilized before the pandemic and the number of remote interactions with a health professional during the pandemic;
- The perception of the health risk of COVID-19 and the intention to be vaccinated when the vaccine against COVID-19 became available.

### 2.3. Statistical Analysis

The software IBM SPSS, v.24 (IBM Corp., Armonk, NY, USA) was used for computing statistical tests. For the categorical variables, absolute and percentage frequencies, and for continuous numerical variables, mean and standard deviation (SD) were calculated. Univariable logistic regression models were developed for two dependent variables reflecting user satisfaction and the ease-of-use of remote visits. For the independent variables included in the regression models, odds ratios (OR), 95% confidence intervals (95% CI), and *p*-values were provided. *p*-values < 0.05 were deemed to be significant.

## 3. Results

### 3.1. Characteristics of the Study Group

The respondent’s mean age (SD) was 40.84 (14.47). In the study sample, 51.16% (*n* = 1233) were females, 34.52% (*n* = 832) were residents of rural areas and 13.82% (*n* = 333) were residents of cities with a population above 500,000, 20.50% (*n* = 494) had lower than secondary education and 28.17% (*n* = 679) had a university education. The mean (standard deviation, SD) HL score was 12.15 (3.70), and the mean (SD) eHL was 25.34 (4.54) in the study group. Detailed characteristics of the study group are shown in Table 1. 

### 3.2. The Use of e-Health Solutions before and during the Pandemic

Before the pandemic, only 18.88% (*n* = 455) adult Internet users had obtained remote physician’s advice based on telephone, VTC, e-mail, or another tool. During the first six months of the COVID-19 pandemic, this percentage increased by 205.27% to 57.63% (*n* = 1389) (Fisher’s exact test, *p* < 0.001). The highest growth of remote advice from physicians was seen in telephone-based communication (344.23%). VTC-based remote visits increased by 51.47% and e-mail-based increased by 60.0%. Among other e-health services, there was a 32.59% increase in e-prescription use (*p* < 0.001) and 35.70% in e-referrals (*p* = 0.005). The use of an electronic booking system for visits to a physician’s office, e-sick leave service, and accessing the Internet patient portal decreased by 19.78%, 13.38%, and 16.24, respectively (Fisher’s exact test for all comparisons *p* < 0.05). Detailed information about the use of e-health services is presented in Table 2.

### 3.3. The Aims of Remote Physician’s Advice during the Pandemic 

The largest part of remote visits were aimed at prescription renewal (43.84%). Only 10.37% (*n* = 144) of the respondents accessed a physician’s teleadvice due to symptoms suggesting a COVID-19 infection. A total of 26.93% (*n* = 374) did so because of other acute symptoms, and 21.45% (*n* = 298) did so as a follow-up or due to the exacerbation of the symptoms of a chronic disease (Table 3). A total of 24.41% (*n* = 339) of respondents contacted a physician to obtain help because of an ill child or another family member. 

### 3.4. Determinants of User Satisfaction with Remote Physician’s Visits

Respondents with higher HL (OR, 95% CI: 1.12, 1.08–1.16) and higher eHL (OR, 95% CI: 1.06, 1.04–1.09) were more likely to be satisfied with remote physician’s advice than those with lower HL and eHL. Additionally, older persons (OR, 95% CI: 1.02, 1.01–1.03), and those living in households with the greatest income (OR, 95% CI: 1.56, 1.04–2.35), were more satisfied than younger ones and those from the lowest income group. Students and vocationally passive persons were less likely to be satisfied than employees. Persons using telephone-based teleadvice were more satisfied than users of other solutions (1.52, 1.07–2.16). Those using teleadvice because of an ill child or another family member were less likely to be more satisfied than others (OR, 95% CI: 0.74, 0.57–0.94) and those who contacted a physician to renew prescriptions were more likely (OR, 95%CI: 1.28 (1.03–1.59) than others. A greater number of types of e-health services used before the COVID-19 pandemic was associated with higher satisfaction with remote visits (OR, 95% CI: 1.20, 1.10–1.31). Satisfaction with remote visits was also significantly associated with the perception of the threat of COVID-19 to health and the intention to be vaccinated against COVID-19. The results of the univariable logistic regression models of user satisfaction and the perception of ease-of-use are provided in Table 4. 

### 3.5. Determinants of the Opinions about Ease-of-Use 

Higher HL, eHL, and older age were associated with the opinions of higher ease-of-use of televisits during the pandemic compared to lower HL, eHL, and younger age (Table 4). Among sociodemographic variables, the ease-of-use of televisits was assessed worse by students and vocationally passive persons than employees, but better by persons with university (Bachelor’s) education than lower than secondary education and by persons with higher income levels than those from the group with the lowest household income. Furthermore, the users of telephone-based televisits were nearly 2.6 times more likely to assess the ease-of-use better than other recipients of televisits (OR, 95% CI: 2.58, 1.82–3.66). Those who used remote visit because of acute symptoms other than COVID-19 were 30% more likely to assess its ease-of-use favorably (OR, 95% CI: 1.34, 1.03–1.75) than those using it for other purposes. The respondents showing a higher perception of the health risk related to COVID-19 and those who had utilized a greater number of e-health services before the pandemic were more likely to assess ease-of-use better than those with a lower feeling of threat and those who had used fewer services. 

## 4. Discussion

In this study, we analyzed the data from a survey performed on a relatively large sample of adult Internet users from Poland. During the first six months of the COVID-19 pandemic, remote physician visits were used by 48% of respondents. The number of e-health services significantly increased during the pandemic; in the case of remote physician visits by 205%, e-referrals by 36%, and e-prescriptions by 33%. Respondents with higher HL or eHL were more likely to express more positive opinions about satisfaction with and the ease-of-use of remote physician’s visits than those with lower HL and eHL. Interestingly, among sociodemographic variables, only age and household income were consistently associated with such opinions. Older persons were more satisfied and better assessed the ease-of-use of remote visits than younger persons. Those declaring higher income were more likely to express higher satisfaction and ease-of-use of e-health services than those with the lowest income. Respondents who had achieved a university education (bachelor’s) were more frequently convinced about the ease-of-use of such services than those with a lower level of education. Finally, high school and university students and vocationally passive persons were less satisfied and expressed more negative opinions about the ease-of-use of e-health services than employees. People using telephone-based remote teleadvice expressed higher satisfaction and ease-of-use of remote visits than people using other tools. Respondents accessing teleadvice because of an ill child or other family members were less satisfied and those using it for renewal of a prescription were more satisfied with e-health services than those using them for other purposes. The perception of the health threat of COVID-19 was significantly associated with a higher assessment of both features of e-health services. Intention to be vaccinated against COVID-19 was significantly associated with higher satisfaction assessment but not ease-of-use. Finally, those who had used more types of e-health services before the pandemic were more likely to assess both characteristics of remote visits positively. 

A narrative review of the deployment of telemedicine in Italy during the COVID-19 pandemic revealed similar phenomena as experienced by users in Poland [34]. The authors of the review reported initial disorientation in the use of telemedicine preceding the preparation of recommendations for its use, an expansion of the telemedicine boundaries, and its high acceptance in the population. In Poland, the first guidelines on how to perform remote physician’s visits were only issued by the Supreme Medical Council four months after the MoH’s announcement recommending the use of telemedicine instead of traditional visits [22]. On the other hand, the first reports showed a high level of satisfaction with remote physician visits among primary care patients [10]. A high level of satisfaction, as high as 82%, was reported by Orrange et al. among internal medicine patients of one of the academic medical centers in the USA [35]. 

Comparing the usage of e-health services between various countries is difficult because it depends on many factors. However, the use rates of remote visits in the first year of the COVID-19 pandemic usually surpass 30–40%. For example, a study carried out in the United Arab Emirates showed that telemedicine services were used during the pandemic by 30.3% of respondents from the general population; 80.8% of them used them to seek physician advice. Usage of telemedicine was significantly associated with older age, female gender, college education, medical insurance, monthly prescriptions, diabetes and immune diseases, and activity on social media [36]. According to Wood et al., early in the pandemic, as many as 50% of HIV clinic visits were substituted by phone-based visits [28].

In general, the results of the Polish study are consistent with the results of the review published by Ashima et al. [37]. Their analysis of 25 studies published between December 2019 and August 2020 revealed high satisfaction with telemedicine encounters among patients with diverse medical conditions. However, according to this review, the age and sex of telemedicine users were not associated significantly with the level of satisfaction. In the Polish population, older age was associated with higher satisfaction and more positive assessment of the ease-of-use of remote visits. This finding could be attributed to several reasons. First, the study was based on the CAWI technique and all respondents were Internet users. So, potentially, other factors than usually reported lower computer- and Internet literacy in the case of older persons shaped the reactions to remote physician visits. As older age is associated with a growing number of chronic medical conditions, the perception of the risk caused by epidemic threats during traditional visits to health care facilities could be very high among older people. Furthermore, as reported earlier, the telephone was the main tool for remote physicians’ visits during the first phase of the pandemic. In this group of respondents, such a mode of communication is usually commonly accepted. Finally, the follow-up visits and renewal of prescriptions for medication taken for longer periods are probably one of the key aims of routine visits to a physician’s office, so the course of such visits is not particularly demanding. It does not require a complicated remote interaction from older patients.

In a study performed in 2020 in the USA, Vosburg & Robinson analyzed the relationship between the characteristics of telemedicine visits and patients’ satisfaction [38]. They found that higher satisfaction was observed among female patients; those who saved more than 30 min due to telemedicine visits, had an easy connection, and used Zoom video visits rather than the telephone only. In our study, gender was not significantly associated with the level of satisfaction. However, the use of telephone-based televisits was associated with a higher assessment of satisfaction and ease of use. Another study performed in the USA showed that the satisfaction of internal medicine patients attending remote visits was significantly associated with fewer technical issues, less concern about privacy or cost, successful face-to-face video communication, income level, trust in the physician, and younger age [35]. 

Many authors have studied the adoption and the acceptance of e-health and telemedicine services during the COVID-19 pandemic among a diversified group of patients. There are also reports analyzing the determinants of the telemedicine services in comparison to people using only in-person visits to a physician’s office. Maietti et al. analyzed the acceptance of telemedicine services among patients with diabetes in Italy during the pandemic [31]. They found that the willingness to continue the use of telemedicine was higher among patients with a higher level of education and those who were unemployed. Older age was a predictor of lower perceived quality of telemedicine and teleassistance. In turn, Brown et al. reported that the predictors of telemedicine vs. in-patient visits among patients with cardiovascular diseases included higher income levels, suffering from coronary arterial disease, hyperlipidemia, and heart failure rather than other conditions [39]. In another study, among parents of children suffering from cardiovascular diseases, the acceptance of televisits during the COVID-19 pandemic was higher among younger persons, those of Hispanic ethnicity, and in the case of children suffering from arrhythmia or acquired heart disease [40]. A nationwide survey among patients with cancer showed that telemedicine was adopted during the pandemic more frequently by younger rather than older patients and those with more comorbidities [41].

Luo et al. retrieved the data from patients’ electronic health records from the Milwaukee area [30]. Videoconferencing visits were used more frequently by younger and white patients and those with private insurance. Telephone visits were used more frequently by older and black patients and those with public insurance. This study revealed that women were more likely than men and persons with college education were more likely than those with lower education to use telemedicine services. 

Sabbir et al. employed the Health Belief Model and the Unified Theory of Acceptance and Use of Technology Model to analyze the antecedents of telemedicine acceptance during the COVID-19 pandemic among generations Y and Z [42]. Satisfaction with telemedicine was treated as an outcome of telemedicine acceptance. According to this model, actual usage behavior, determined by the actual usage frequency, was positively associated with satisfaction with telemedicine [42]. It is of interest that Rahi et al. developed a complex model of patient attitude toward the adoption of telemedicine health services integrating the unified theory of acceptance and use of technology (UTAUT), the protection motivation theory (PMT), and the information success model [43]. According to these authors, their model explained as much as 80% of the variance in patient attitude. 

### Limitations

The analysis was based on data from a survey performed among Internet users. This is a major limitation of this study as one can assume that respondents were more computer-literate, and, as a result, their acceptance of e-health services was higher. The opinions of Internet non-users could not be analyzed with the CAWI technique.

The study is based on responses to a questionnaire consisting of 55 items, including two validated instruments for assessing HL and eHL. Therefore, there was limited space for measuring the key constructs analyzed in the paper and applying a more complex conceptual framework stemming from the technology acceptance models. User satisfaction and ease-of-use were assessed based on singular items only. So, on one hand, we could evaluate the role of a relatively large number of determinants of user experience. On the other hand, the differentiation between the users’ responses to relevant items was limited.

Although patients were offered many e-health functionalities during the pandemic, we had to focus on assessing their experience with the selected functionalities. As a remote physician visit was a novelty for most patients, we decided to focus on with this type of e-health functionality. 

The study was carried out in the relatively early phase of the COVID-19 pandemic. Unfortunately, with a cross-observational design, one cannot assess the trends in analyzed phenomena. A high user acceptance of remote physician’s visits could be only an initial reaction to unexpected epidemic threats, likely to change with later phases of the pandemic.

Finally, the results of the analysis could hardly be extrapolated to other countries, differing from Poland in the characteristics of their health care systems and measures implemented during the pandemic.

## 5. Conclusions

The COVID-19 pandemic resulted in a significant increase in the use of remote advice from physicians in Poland. The satisfaction with remote visits and the opinions about their ease-of-use depends on many factors. Higher HL and eHL, older age, telephone-based teleadvice, more intense use of e-health services before the pandemic, and higher perception of the threat of COVID-19 were consistently significant predictors of higher satisfaction and more positive assessment of the ease-of-use of remote visits. Higher income and being employed were also associated with higher satisfaction and a more favorable assessment of ease-of-use. In turn, the intention to be vaccinated against COVID-19 was significantly associated with higher satisfaction. Unexpectedly, older persons more positively assessed the use of e-health services than younger persons. Furthermore, the assessment of e-health services did not depend on the place of residence and only depended to a limited extent on respondents’ education level.

The findings of this study can guide the further implementation of e-health services in Poland. First of all, the traditional perception of the relationship between sociodemographic variables and the acceptance of health-related technologies cannot be taken for granted. In this Polish study, neither gender, place of residence, nor marital status played a role in user satisfaction or ease-of-use of remote physician visits. It was also not expected that the importance of the level of education would be limited. Unexpectedly, older respondents showed a more accepting attitude. It also seems that the aim of the given e-health services used may be a predictor of user satisfaction. Finally, health and e health literacy should be considered when the society is offered new modes of delivery of health services. The role of health literacy in reactions to e-health services can be perceived as a by-product of the general capacity to deal with health issues. In practical terms, the nationwide implementation of e-health solutions should be preceded by appropriate recognition of general computer and Internet literacies. One could also assume that harmonized actions focused on improving health and e-health literacies will lead to better handling of health-related information available from different sources and more efficient use of available health care services.

## Figures and Tables

**Table 1:** Characteristics of the study group.

Variable	Variable Categories	All Respondents (*n* = 2410)		Users of Remote Visits (*n* = 1389)	
		%	*n*	%	*n*
Gender	female	51.16	1233	55.65	773
	male	48.84	1177	44.35	616
Place of residence	rural	34.52	832	35.49	493
	urban below 20,000 inhabitants	9.54	230	9.94	138
	urban 20,000–100,000 inhabitants	23.44	565	22.17	308
	urban 100,000–200,000 inhabitants	9.46	228	10.37	144
	urban 200,000–500,000 inhabitants	9.21	222	9.14	127
	urban above 500,000 inhabitants	13.82	333	12.89	179
Education	lower than secondary	20.50	494	20.16	280
	secondary	39.50	952	38.80	539
	post-secondary non-university	11.83	285	12.38	172
	university Bachelors	11.78	284	11.88	165
	university Masters	16.39	395	16.77	233
Net monthly household income	not more than 1000 PLN	11.74	283	11.23	156
	1001–1500 PLN	17.80	429	17.57	244
	1501–2000 PLN	22.66	546	23.40	325
	2001–3000 PLN	28.76	693	29.01	403
	more than 3000 PLN	19.05	459	18.79	261
Vocational status	employee	49.50	1193	52.12	724
	self-employed or farmer	8.17	197	8.57	119
	retired or on disability pension	12.41	299	12.81	178
	high school or university student	10.91	263	7.92	110
	vocationally passive incl. unemployed	19.00	458	18.57	258
Marital status	married or in partnership	67.01	1615	71.20	989
	single	21.70	523	17.64	245
	widowed	3.82	92	3.53	49
	divorced or in separation	7.47	180	7.63	106
The perception of the COVID-19 health threat	decidedly no	7.6	184	6.1	85
	no	11.0	264	10.2	141
	difficult to say	22.2	534	22.1	307
	yes	29.4	709	28.9	401
	decidedly yes	29.8	719	32.8	455
Intention to be vaccinated against COVID-19	decidedly no	26.5	638	24.0	334
	no	12.5	302	13.3	185
	difficult to say	33.7	813	32.8	456
	yes	14.1	340	15.6	216
	decidedly yes	13.2	317	14.3	198
Satisfaction of remote visit	satisfied			58.94	811
	not satisfied or difficult to say			41.06	565
Ease-of-use of remote visit	easy to use			67.73	934
	difficult to use or difficult to say			32.27	445

**Table 2:** The use of e-health services before and six months after the beginning of the COVID-19 pandemic.

Variable	Before COVID-19 Pandemic		First 6 Months of the Pandemic		% Change in the Service Use	*p* *
	*n*	%	*n*	%	%	
Remote visit						
irrespective of the tool	455	18.88	1389	57.63	205.27	**<0.001**
telephone-based	280	11.62	1244	51.62	344.23	**<0.001**
VTC-based	82	3.40	124	5.15	51.47	**0.003**
e-mail-based	118	4.90	189	7.84	60.00	**<0.001**
Other e-health services						
e-booking of visits to a physician	664	27.55	532	22.07	−19.78	**<0.001**
e-prescription	1019	42.28	1351 ^#^	56.06	32.59	**<0.001**
e-sick leave	463	19.21	401 ^#^	16.64	−13.38	**0.022**
e-referral	289	11.99	392 ^#^	16.27	35.70	**<0.001**
Internet patient portal	475	19.71	398	16.51	−16.24	**0.005**

* Fisher exact test, ^#^ the number of e-health services provided both during remote and traditional visits to physicians’ offices. Significant *p*-values were bolded.

**Table 3:** The aims and modes of the televisits during the COVID-19 pandemic (*n* = 1389).

Aim	*n*	%
Symptoms suggesting COVID-19	144	10.37
Other acute symptoms	374	26.93
Follow-up or exacerbation of chronic disease	298	21.45
Ill child or other family member	339	24.41
Prescription renewal	609	43.84

**Table 4:** Univariable logistic regression for user satisfaction and ease-of-use of remote physician visits during the COVID-19 pandemic.

Variables	Categories	User Satisfaction		Ease-of-Use	
		OR (95% CI)	*p*	OR (95% CI)	*p*
HL		1.12 (1.08–1.16)	**<0.001**	1.18 (1.14–1.22)	**<0.001**
eHL		1.06 (1.04–1.09)	**<0.001**	1.09 (1.06–1.11)	**<0.001**
Age		1.02 (1.01–1.03)	**<0.001**	1.01 (1.009–1.02)	**0.017**
E-health services before the pandemic ^$^		1.20 (1.10–1.31)	**<0.001**	1.21 (1.12–1.30)	**<0.001**
Gender	Female ^#^				
	male	1.19 (0.96–1.47)	0.121	0.98 (0.78–1.23)	0.864
Place of residence	rural ^#^				
	urban below 20,000	0.78 (0.53–1.14)	0.197	0.83 (0.56–1.24)	0.363
	urban 20,000–100,000	0.98 (0.73–1.31)	0.891	1.07 (0.78–1.45)	0.682
	urban 100,000–200,000	0.88 (0.60–1.28)	0.505	0.84 (0.57–1.25)	0.396
	urban 200,000–500,000	0.95 (0.64–1.42)	0.820	0.98 (0.65–1.49)	0.926
	urban above 500,000	0.97 (0.69–1.38)	0.886	1.15 (0.79–1.67)	0.476
Education	lower than secondary ^#^				
	secondary	0.97 (0.73–1.31)	0.857	1.04 (0.77–1.42)	0.786
	post-secondary non-university	1.13 (0.77–1.67)	0.541	1.05 (0.70–1.57)	0.809
	university Bachelors	1.40 (0.94–2.09)	0.101	1.67 (1.08–2.58)	0.020
	university Masters	1.15 (0.81–1.65)	0.428	1.31 (0.90–1.91)	0.161
Net monthly income per household member	≤1000 PLN ^#^				
	1001–1500 PLN	1.56 (1.04–2.36)	**0.032**	1.71 (1.13–2.61)	**0.012**
	1501–2000 PLN	1.27 (0.87–1.88)	0.219	1.78 (1.20–2.66)	**0.004**
	2001–3000 PLN	1.31 (0.90–1.90)	0.161	1.83 (1.24–2.68)	**0.002**
	>3000 PLN	1.56 (1.04–2.35)	**0.030**	1.34 (0.89–2.01)	0.161
Vocational status	Employee ^#^				
	self-employed or farmer	0.87 (0.59–1.29)	0.488	0.88 (0.57–1.34)	0.541
	retired or on disability pension	1.34 (0.94–1.90)	0.103	0.89 (0.62–1.28)	0.537
	high school or university student	0.51 (0.34–0.77)	**0.001**	0.49 (0.32–0.74)	**0.001**
	vocationally passive incl. unemployed	0.69 (0.52–0.92)	**0.012**	0.53 (0.39–0.71)	**<0.001**
Marital status	married or in partnership ^#^				
	single	0.86 (0.65–1.14)	0.288	0.99 (0.73–1.34)	0.963
	widowed	0.81 (0.45–1.45)	0.475	0.97 (0.53–1.80)	0.933
	divorced or in separation	1.26 (0.83–1.91)	0.285	0.92 (0.60–1.40)	0.693
VTC-based RPA	No ^#^				
	Yes	1.38 (0.94–2.04)	0.104	0.91 (0.62–1.35)	0.641
Telephone-based RPA	No ^#^				
	Yes	1.52 (1.07–2.16)	**0.019**	2.58 (1.82–3.66)	**<0.001**
E-mail-based RPA	No ^#^				
	Yes	1.14 (0.83–1.56)	0.426	0.83 (0.60–1.15)	0.263
COVID-19 symptoms	No ^#^				
	Yes	0.93 (0.65–1.32)	0.682	0.83 (0.58–1.18)	0.298
Other acute symptoms	No ^#^				
	Yes	1.08 (0.84–1.37)	0.558	1.34 (1.03–1.75)	**0.027**
Follow-up or exacerbation of chronic disease	No ^#^				
	Yes	1.13 (0.87–1.47)	0.355	0.80 (0.61–1.04)	0.098
Ill child or another family member	No ^#^				
	Yes	0.74 (0.57–0.94)	**0.015**	0.93 (0.72–1.21)	0.599
Prescription renewal	No ^#^				
	Yes	1.28 (1.03–1.59)	**0.026**	1.24 (0.99–1.56)	0.066
The perception of the COVID-19 health threat	decidedly no ^#^				
	No	1.01 (0.59–1.75)	0.964	1.70 (0.97–2.97)	0.063
	difficult to say	0.78 (0.48–1.27)	0.325	1.24 (0.76–2.02)	0.393
	yes	1.13 (0.70–1.82)	0.610	1.62 (1.009–2.61)	**0.049**
	decidedly yes	1.82 (1.13–2.93)	**0.013**	2.31 (1.43–3.74)	**0.001**
Intention to vaccinate against COVID-19	decidedly no ^#^				
	No	1.11 (0.77–1.59)	0.582	0.77 (0.53–1.13)	0.186
	difficult to say	1.38 (1.04–1.84)	**0.027**	1.05 (0.77–1.42)	0.760
	Yes	1.35 (0.95–1.91)	0.094	0.93 (0.65–1.34)	0.704
	decidedly yes	1.89 (1.31–2.74)	**0.001**	1.12 (0.76–1.64)	0.575
Number of episodes *	1 ^#^				
	2–3	0.80 (0.59–1.07)	0.124	0.88 (0.65–1.19)	0.402
	4–5	1.03 (0.72–1.47)	0.859	1.18 (0.81–1.71)	0.388
	>5	0.85 (0.57–1.27)	0.430	1.09 (0.71–1.68)	0.682

^#^ referential category of independent variable; ^$^ the number of types of e-health services used before the COVID-19 pandemic, * the number of episodes of remote contact with a health professional during the pandemic. Significant *p*-values were bolded.

## Data Availability

The data are not publicly available due to privacy and ethical restrictions. The authors did not include in the information about the study provided to the participants that the public access to the data obtained during the survey may be considered. Access to the data will be granted on a case-by-case basis on a justified request after receiving consent from the Bioethical Committee at Jagiellonian University.
